# Quantitative Ultrasound for the Assessment of Bone Quality in Hyperphenylalaninemia/Phenylketonuria Patients: Vitamin D Supplementation Versus No Supplementation

**DOI:** 10.3390/metabo15110754

**Published:** 2025-11-20

**Authors:** Albina Tummolo, Giada De Ruvo, Marta Di Nicola, Vito Di Tullio, Livio Melpignano, Donatella De Giovanni, Rosa Carella

**Affiliations:** 1 Children Hospital Giovanni XXIII, Department of Inherited Metabolic Disorders and Clinical Genetics, Azienda Ospedaliero-Universitaria Consorziale, 70126 Bari, Italy; giadaderuvo1@gmail.com (G.D.R.); vditullio11@libero.it (V.D.T.); donatella.degiovanni@policlinico.ba.it (D.D.G.); rosa.carella@policlinico.ba.it (R.C.); 2 Laboratory of Biostatistics, Department of Medical, Oral and Biotechnological Sciences, University G. D’Annunzio Chieti-Pescara, 66100 Chieti, Italy; marta.dinicola@unich.it; 3 Medical Direction, Giovanni XXIII Children Hospital, Azienda Ospedaliero-Universitaria Consorziale, 70126 Bari, Italy; livio.melpignano@policlinico.ba.it

**Keywords:** phenylketonuria, hyperphenylalaninemia, quantitative ultrasound, QUS, bone quality, micronutrients, vitamins, supplementation

## Abstract

**Background/Objectives:** Skeletal impairment has been reported as a common finding in Hyperphenylalaninemia (HPA)/Phenylketonuria (PKU) patients regardless of age and method of diagnosis, both in children and adults. Quantitative Ultrasound (QUS) is a radiation-free and low-cost method for assessing bone quality, used in various chronic conditions. **Methods:** Bone quality was evaluated using a calcaneal QUS device. Auxological parameters, nutritional intakes, and plasma levels of key bone biomarkers were also registered. The population was divided into four groups: PKU patients under diet therapy and HPA patients on a free diet, both divided into receiving or not receiving single vitamin D supplementation. **Results:** All HPA/PKU patients had median bone quality index (BQI) Z- and T-score values lower than −1, with slightly better values in HPA children and PKU-supplemented adults. Dietary vitamin D intake in PKU patients was significantly higher than in HPA subjects (*p* < 0.001), due to protein substitute supplementation. However, plasma 25(OH) vitamin D levels, although increased compared to baseline, were still overlapping among groups (*p* = 0.845) after supplementation. Approximately a quarter of both pediatric and adult non-supplemented PKU patients had Z-score and T-score levels below −2, and this percentage decreased with vitamin D supplementation in all groups. In PKU-supplemented patients, the Broadband Ultrasound Attenuation (BUA) was significantly higher than in the other groups (*p* = 0.040). **Conclusions:** The improvement in BUA may represent preliminary evidence of the effect of vitamin D on bone architecture, which could encourage this supplementation to prevent the worsening of bone structure and reduce the risk of fractures.

## 1. Introduction

Phenylketonuria (PKU, ORPHA79254, MIM 261600) is an inherited metabolic disorder caused by mutations in the Phenylalanine Hydroxylase (PAH) gene, coding the enzyme responsible for the metabolization of Phenylalanine (Phe) into Tyrosine (Tyr). As a consequence, Phe accumulates in the blood tissues and is transported across the blood–brain barrier. Its high concentration can lead to intellectual disability, behavioral abnormalities, and systemic issues in the life-long PKU follow-up [1,2].

Both PKU and mild HPA are caused by pathogenic variants in the PAH gene, leading to reduced activity of phenylalanine hydroxylase. The residual enzymatic activity determines the biochemical phenotype: PKU patients show markedly elevated blood phenylalanine concentrations and require a lifelong hypoproteic diet supplemented with amino acid mixtures, while HPA patients exhibit only mild elevation of phenylalanine levels and can maintain a normal diet.

In this context, low bone mineral density (BMD) has been described in both early-diagnosed and late-diagnosed Phenylketonuria (PKU) patients [3]. Adults with PKU showed significantly reduced BMD Z-Scores compared to the general population, although without a higher fracture risk [4]. Also, in pediatric patients with PKU, evidence regarding bone density status demonstrates lower than normal reference ranges [5,6], although without a clear correlation with the common determinants of osteopenia/osteoporosis in childhood [7].

Although traditionally it has been hypothesized that reduced intake of calcium and phosphorus from natural foods represents the main cause of decreased bone mass in patients with PKU, recent studies indicate that blood calcium status in treated patients is often normal or even higher than in the general population [8,9]. In our recent analysis, we also observed a tendency toward calcium over supplementation through protein substitutes, particularly in formulations designed for children over three years of age [10]. At the same time, it has been proposed that the composition of amino-acid-based medical foods—used in the dietary management of PKU—imposes an increased systemic acid load with consequent elevated urinary excretion of calcium and magnesium, a factor potentially relevant in the genesis of osteopenia/osteoporosis in these subjects [11]. On the contrary, it has been documented that patients with PKU are more often vitamin D sufficient than the general population [12] and lower than normal BMD values have also been found in Hyperphenylalaninemia (HPA) patients, who should be protected by a free diet regimen [13].

In the PKU diet, micronutrient supplementation happens mainly through amino acids (AA) mixtures, which are enriched with vitamins and oligoelements to supply the poor intake by diet [14,15]. Adequate intake, however, is not always reached, because physiological vitamin status is more dependent on natural protein intake than on their content as supplementation of AA formulas, and therefore single micronutrient supplementation is often necessary to reach physiological status [16]. However, studies on the effect of single vitamin D supplementation in this group of patients are rare and often focused more on the assessment of change in vitamin D plasma levels than on the effect on bone health status [17,18,19].

Quantitative ultrasound (QUS) has been reported in previous studies as a method to assess bone quality in children. In QUS, the attenuation and speed of propagation of the ultrasound wave provide information on the physical properties of bone [20]. Advantages of QUS are as follows: no ionizing radiation, lower cost, high portability, and lower time spent [21]. For these reasons, its clinical applications have recently been addressed by the International Society of Clinical Densitometry (ISCD), 2007 Pediatric Position Development Conference [22]. In particular, calcaneal QUS is considered the standard parameter for QUS study of bone health status and has been used in children and adolescents to assess osteopenia and fracture rate in subjects with bone and mineral disorders, in several chronic disorders [23,24].

In HPA/PKU patients, QUS has been rarely used as a method to follow up skeletal involvement. Evidence on bone status report lower values than the general population [25,26], but the effect of a hypoproteic diet has not been clearly elucidated, as osteopenia was also detected from an early age in PKU children, without a clear relationship to diet therapy [27]. Furthermore, the role of vitamin D supplementation in the context of multivitamin supplementation through AA mixtures remains a topic of discussion.

In this study, we aimed to assess QUS parameters in both free-diet HPA patients and PKU patients on a hypoproteic and amino-acid-supplemented diet, and to evaluate the effect of single vitamin D supplementation on those parameters.

## 2. Materials and Methods

We conducted a retrospective cross-sectional study on both PKU patients (Phe levels  ≥  360 umol/L) and HPA patients (120 umol/L  >  Phe  <  360 umol/L). Data were collected between April and June 2025 at the Giovanni XXIII Pediatric Hospital. This study was purely observational and non-interventional. No experimental assignment or intervention was applied by the authors.

### 2.1. Patients

Inclusion criteria were as follows: patients with a confirmed diagnosis of either HPA or PKU who had undergone a QUS for bone health assessment over the follow-up were included in the study. Healthy control data were not available because of the retrospective design. Instead, QUS results were interpreted using validated age- and sex-specific reference ranges from the manufacturer and published pediatric/adult normative data, which serve as an internal reference comparable to a normal control population. The total study population was categorized based on clinical management and supplementation into four groups: PKU patients under diet therapy and AA mixtures, those also receiving single vitamin D supplementation, and patients with HPA on a free diet, with and without vitamin D supplementation. A supplementation period of three months was considered the minimum length to be included in the study, with a maximum duration of six months. Supplementation commenced in all cases of vitamin D levels < 20 ug/L, and the dose of supplementation was 400 UI/day [28].

Exclusion criteria were as follows: the absence of QUS measurement/results and incomplete records of laboratory tests at the time of ultrasound examination. Also, concomitant chronic disorders that could interfere with the patients’ bone status were considered as exclusion criteria.

### 2.2. Measurements

For each participant, anthropometric data, including weight and height to calculate body mass index (BMI), were collected at the time of the QUS. Protocol of assessment was the same as described in Tummolo et al. [29]. Anthropometric measurements were conducted using the InBody 230 analyzer (Biospace Corp., Seoul, Republic of Korea). Bone quality was evaluated using the SONOST 3000 (Osteosys, Seoul, Korea), a calcaneal QUS device which enabled the calculation of both the bone quality index (BQI) and its corresponding standard deviation score (SDS). According to the ISCD Pediatric Official Position (Z-score of −2.0 or lower was defined as below the expected range for age, and a Z-score above −2.0 was defined as within the expected range for age) [7]. The BQI was determined using two acoustic parameters: broadband ultrasound attenuation (BUA), expressed in dB/MHz, and speed of sound (SOS), expressed in m/s. BUA reflects bone microarchitecture and is sensitive to temperature variations, while SOS is suggestive of bone mineral density and inversely influenced by temperature [30]. The BQI was obtained by applying correlation coefficients (α, β) to an arithmetic formula, as described in a previous study by our group [13]. All QUS assessments were performed using the same equipment and standardized protocols across the entire patient cohort, ensuring consistency and reliability of the data. According to ISCD Pediatric Official Position, validated heel QUS devices predict fragility fracture in postmenopausal women and men over the age of 65 independently of central DXA BMD [7].

### 2.3. Laboratory Data

Laboratory data, including serum calcium and 25(OH) vitamin D, were obtained from routine blood tests performed at the time of the QUS. To assess dietary compliance, additional parameters, such as plasma Phe and Tyr concentrations, the Phe/Tyr ratio, and total serum proteins, were analyzed. All biochemical analyses were carried out at the same clinical laboratory to ensure reproducibility. High-performance liquid chromatography (HPLC) was used to measure plasma Phe and Tyr levels. The reference range for laboratory tests was determined according to the valid laboratory kit.

### 2.4. Nutritional Assessment

A nutritional analysis was conducted in patients with PKU undergoing dietary treatment with the addition of protein substitute supplementation and in HPA patients on a free-diet regimen. The intake of key bone-related nutrients, such as total proteins, vitamin D, calcium, and phosphorus, was examined, considering those provided by natural foods and those derived from protein substitutes. For this reason, patients were asked to complete a 3-day food diary. The Winfood Pro software (version 3.0.0, 2011, Medimatica Srl, Teramo, Italy) was used for intake calculations.

### 2.5. Statistical Methods

Descriptive statistics for continuous variables are reported as median and interquartile range (IQR), given the non-normal distribution of most variables, as assessed using the Shapiro–Wilk test. For inferential analysis, non-parametric tests were employed. The Mann–Whitney U test was used to compare two independent groups, while the Kruskal–Wallis test was applied for comparisons involving more than two groups. When significant differences were found with the Kruskal–Wallis test, post hoc pairwise comparisons were conducted using the correction to control for multiple testing. Associations between ordinal or non-normally distributed continuous variables were analyzed using Spearman’s rank correlation coefficient (ρ). All statistical tests were two-tailed, and *p*-values < 0.05 were considered statistically significant. Statistical analyses were performed using Jamovi (version 2.6.45).

## 3. Results

A total of 110 patients were included in the analysis: 16 with mild hyperphenylalaninemia (HPA), 6 with HPA receiving supplementation (HPA + SUP), 74 with PKU, and 14 with PKU receiving supplementation (PKU + SUP). The overall median age was 16 years (IQR: 9–24), with no significant differences among groups (*p* = 0.116). Of the total patients, 62% were younger than 18 years, and females were slightly more represented (61.8%), with no statistically significant differences in sex distribution (*p* = 0.326). Median body mass index (BMI) values were also comparable across groups (*p* = 0.086) (Table 1).

Sixty-four PKU patients (86%) were administered with AA mixtures and four (5%) took glycomacropeptides (GMP). The nutritional intake by protein substitutes for the PKU group is reported in Table 2. No statistically significant differences were found for protein equivalent, calcium, phosphorus, and vitamin D intakes between the two groups of PKU patients. Considering intakes from natural foods, natural protein, calcium, phosphorus, and vitamin D intake, were instead significantly lower in PKU groups than in HPA groups (Table 2).

**Figure 1:**
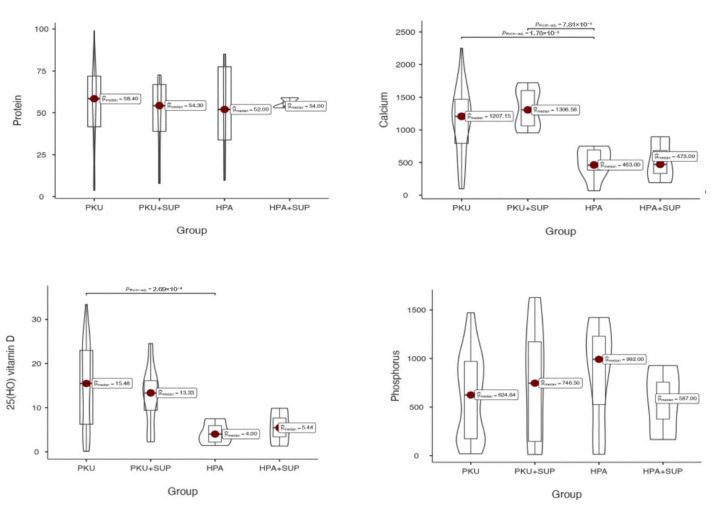
Total nutrient intakes in the study groups (PKU, PKU with supplementation [PKU + SUP], HPA, and HPA with supplementation [HPA + SUP]). Violin plots display the distribution of intakes for protein (**top left**, 0 = 0.831), calcium (**top right**, *p* = 0.052), 25(OH) vitamin D (**bottom left**, *p* = 0.001), and phosphorus (**bottom right**, *p* = 0.713). Red dots indicate the median values, with boxes showing the interquartile range. *p*-values refer to pairwise group comparisons.

When considering the total intake of nutrients from both protein substitutes and natural foods, calcium and vitamin D intakes become significantly higher in PKU patients than in HPA patients (*p* = 0.05; *p* = 0.001, respectively), due to the contribution from protein substitutes. In contrast, total protein intake becomes overlapping between the two groups, as well as phosphorus intake. (Figure 1).

Biochemical parameters among the four patient groups, and normal values are reported in Table 3. Calcium levels fell in all cases within the normal range and were highest in the HPA + SUP group, although this difference did not reach statistical significance. The median 25(OH)Vitamin D levels were in all cases higher than 20 ng/mL, with the upper limit of the range reaching the sufficiency levels (>30 ng/mL) in all groups but in the HPA + SUP group (*p* = 0.8). Phe levels and Phe/Tyr ratio were significantly different, and tyrosine level was comparable among groups (*p* = 0.576).

**Figure 2:**
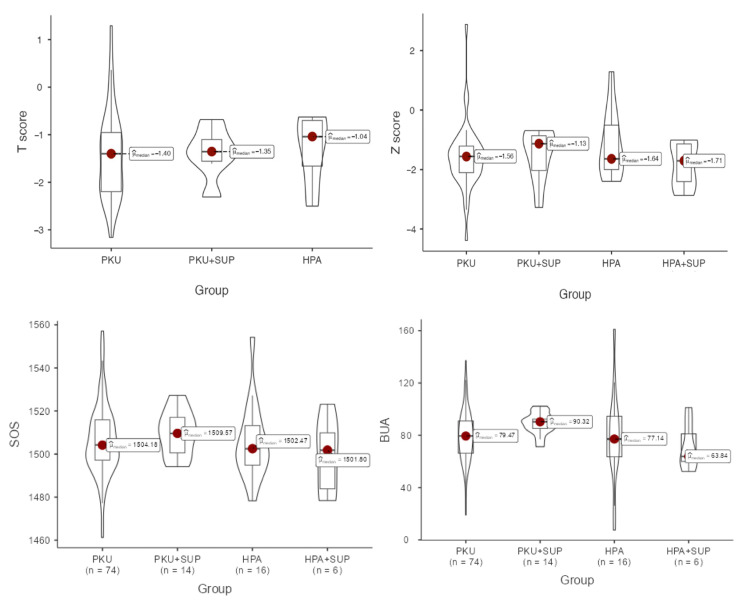
Comparison of data distributions across groups using box and violin plots. For each group, the box plot displays the median (horizontal line) and the interquartile range (represented by the box limits). The accompanying violin plots display the estimated probability density of the data, allowing for a visual comparison of distributional patterns between groups. T-scores were used for adults (**top left**, *p* = 0.889), while Z-scores were used for children (**top right**, *p* = 0.577), to account for age-related differences in normative data. SOS (**bottom left**, *p* = 0.580) and BUA (**bottom right**, *p* = 0.040) were shown.

Bone quality assessment results were as follows: bone mineral density, expressed as a T-score in adults and a Z-score in children, did not differ significantly across the four groups. In adults, the median T-score in the overall population was –1.40 (IQR –2.18 to –0.93). Patients with HPA had the highest values (median –0.82, IQR –1.20 to –0.58), whereas patients with PKU showed lower scores (median –1.47, IQR –2.22 to –1.04), with PKU + SUP patients presenting intermediate values (median –1.35, IQR –1.55 to –1.10). Despite this apparent gradient, differences did not reach statistical significance (Kruskal–Wallis *p* = 0.889). In children, the overall median Z-score was –1.54 (IQR –2.11 to –1.04). Values were similar across groups, ranging from –1.71 (IQR –2.41 to –1.14) in HPA + SUP patients to –1.13 (IQR –2.03 to –0.86) in PKU + SUP patients, with no significant differences (*p* = 0.577). Thus, although bone density indices tended to be slightly better in HPA patients and lower in PKU patients, variability within groups prevented these trends from achieving statistical significance (Figure 2).

Distributions of all the available QUS output values are reported in Table 4. BQI was higher in the PKU supplemented group, although without significant difference compared to other groups. BUA was significantly higher in the above group (*p* = 0.040), whereas it showed the lowest levels in the HPA-supplemented group. Z-score reached the highest levels in HPA children, and the T-score in PKU-supplemented adults.

In adult patients, when applying the <−2 threshold, the prevalence of PKU patients falling below this cut-off level was 35% (n = 11). In contrast, only 17% of PKU + SUP and 20% of HPA adult patients presented T-score levels below −2. No HPA patients supplemented with vitamin D had a T-score level under −2.

**Figure 3:**
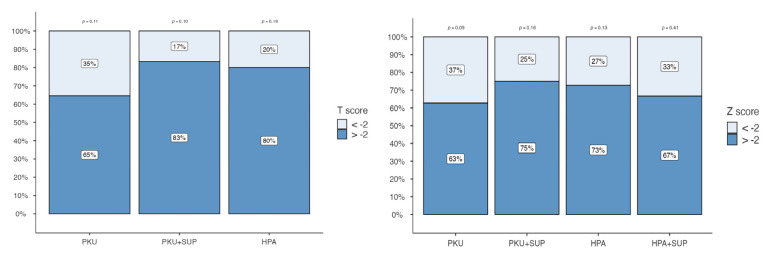
Percentage distribution of T-scores (left panel) and Z-scores (right panel) across patient groups: PKU, PKU with supplementation (PKU + SUP), HPA, and HPA with supplementation (HPA + SUP). Bars represent the proportion of subjects with values below −2 (light blue) and above −2 (dark blue). Percentages are shown within each bar segment. *p*-values reported above the columns indicate statistical comparisons between groups.

In pediatric patients, the Z score < −2 threshold was observed in 37% of the PKU sample (n = 16), while smaller proportions were found in PKU + SUP (25%), HPA (27%), and HPA + SUP (33%) (Figure 3). Spearman’s rank correlation coefficient did not reveal any significant correlation with total protein, vitamin D, phosphorus, calcium intake, gender, age, or BMI. Notably there was no correlation of QUS parameters with Phe levels and patients falling within the target Phe levels according to European guidelines did not result in better bone quality (73.9 vs. 70.6; *p* = 0.724).

## 4. Discussion

There is a long-standing concern that bone health in HPA/PKU patients is poorer than that of the general population [3,5], due to the diet regimen, potentially leading to growth failure and fractures, and to the hyperphenylalaninemia itself [31]. However, studies conducted to date have produced conflicting findings in terms of bone mineral density in this group of patients, possibly due to differences in the age of the patients studied, type of HPA considered, methods of assessment, and the criteria applied.

It is increasingly acknowledged that the development of the osteoporotic state involves the interaction of multiple mechanisms. Understanding the pathogenesis of osteoporosis is closely linked to understanding the pathophysiology of hyperphenylalaninemia in the human body.

The dietary protein content has been largely involved in maintaining a satisfactory bone health status. Studies on children fed with a vegetarian diet, similar to the PKU diet pattern, suggest that the quality of protein in vegan diets is controversial for bone health, due to the pattern of amino acids. Of note, the so-called protein digestibility corrected amino acid score [32] of most plant proteins is lower than that of animal proteins. Studies confirm lower intakes of vitamin D in such a diet pattern and conclude that an adequate supply of this vitamin is significant in a plant-based diet [33].

In this context, the hypoproteic diet in PKU may be involved in the pathogenesis of osteopenia, due to reduced bone-forming substrates. However, osteopenia also occurs in patients who have never received dietary therapy, such as HPA patients. Humans and animal studies find no correlation between bone formation and resorption markers and plasma hyperphenylalaninemia [18,34]. Evidence on mouse models showed a mesenchymal stem cell developmental defect in the osteoblast pathway, secondary to energy dysregulation and oxidative stress, a well-known pathological mechanism linked to hyperphenylalaninemia [35].

In our study, all HPA/PKU patients had median BQI Z- and T-score values lower than −1, with slightly better values in HPA children and PKU-supplemented adults. Dietary vitamin D intake in PKU patients was significantly higher than in HPA subjects, likely due to supplementation by amino acid mixtures [36]. Despite that, plasma 25(OH) vitamin D levels were comparable among groups.

The necessity of vitamin D supplementation in the PKU diet has not been clarified yet, and supplementation is generally prescribed based on the vitamin status, regardless of the vitamin D intake via amino acid mixtures. The vitamin status assessment in studies on PKU patients demonstrates that in many cases, vitamin levels were higher than those of the general population, with vitamin D deficiency ranging from 35% of patients [31] to 53.57% of the population considered [16]. In a systematic review with meta-analysis, vitamin D levels in PKU patients did not significantly differ from those of healthy controls [37].

Although no differences were found in our sample across groups regarding vitamin D levels, when assessing bone quality, approximately a quarter of both pediatric and adult non-supplemented PKU patients had Z-score and T-score levels below −2. This percentage decreased in the case of vitamin D supplementation. This difference was not noticeable for the HPA group. Furthermore, in PKU-supplemented patients, the Broadband ultrasound attenuation, one of the QUS parameters measuring the differential attenuation of sound waves transmitted through the bone, was significantly higher than in the other groups.

In recent years, this technique of quantitative assessment of the skeleton has been developed as an alternative method to bone mass density assessment for the study of bone quality and as a predictor of fracture risk, focusing on the assessment of bone morphology and microarchitecture [38].

BUA has been considered an index of hip fractures in the osteoporosis-risky population, such as postmenopausal women. In a recent study on 6189 postmenopausal women older than 65 years, QUS was performed and compared with the results of bone mineral density measurements of the calcaneus and hip. BUA was associated with a doubling of the risk for hip fractures in an overlapping way with calcaneus BMD [39].

Calcaneal bone mineral density and BUA were also compared in 236 patients, including 77 with osteoporotic fractures. BUA showed the most significant difference between the two populations and discriminated between fractures and nonfracture subjects [40]. Finally, BUA has also been implicated as a potential marker to stratify individuals at risk of deterioration in physical health [41].

Despite the increasing use of quantitative ultrasound techniques in bone assessment, the vast majority of bone health assessments in HPA/PKU patients are still based on BMD measurements, both in adults and children. In a recent study on 18 adults with PKU and matched controls, a reduced bone mineral density T-score was detected in patients [18], although the vast majority were vitamin D sufficient. Neither Phe blood concentration nor dietary habits or lifestyle were associated with BMD in regression analysis. A metanalysis by Rocha on 4097 PKU adults highlighted that mean BMD Z-scores were statistically significantly lower compared with an age-matched control or reference population, but still within the expected range for age (>−2.0). The estimated prevalence of BMD Z-scores ≤−2.0 was 8%. However, the small number of observations in the subgroup analyses limited interpretation.

Male sex and low BMI conferred a higher risk for low BMD in classic PKU, whereas Phe levels and dietary adherence do not, in a study on PKU adults [42]. Compliance to hypoproteic diet with more proteins from AA supplements assumption and low BMI were associated with low BMD in another study [43].

In the pediatric setting, a case series on 48 children who took either amino acid (L-AA) or casein glycomacropeptide substitutes (CGMP-AA) as their main protein source [19], reports that bone density was clinically normal, although the median Z-scores were below the population mean. Blood biochemistry was within the reference ranges, with no advantage to bone density observed from taking CGMP-AA compared with L-AAs. In our sample, only a few patients were administered with GMP (n = 4); therefore, a comparison between the two types of protein substitutes was not possible.

This study has some strengths and some limitations. It provides evidence on QUS parameters on a relatively large sample of patients and associates, and, for the first time, single vitamin D supplementation with parameters related to bone structure and architecture. However, due to its retrospective design, group sizes were not balanced. This imbalance, reflecting the real-world distribution of cases and not influenced by predefined allocation criteria, may have affected the statistical power of some comparisons and could increase the risk of Type II error. In this context, the use of non-parametric tests, which are robust to unequal group sizes and non-normal distributions, partially mitigates this issue. Finally, as with all retrospective studies, causality cannot be firmly established, as temporal relationships between exposures and outcomes are more difficult to verify in this type of study.

## 5. Conclusions

Although there is still no unanimous consensus on how to assess bone health in HPA/PKU patients [44], a regular skeletal evaluation should be part of the follow-up of both children and adults affected by this metabolic disorder. The increasing evidence on the capability of QUS parameters, such as BUA, in evaluating the bone architecture and the fracture risk should facilitate the introduction of this low-cost and radiation-free technique in the instrumental evaluation of bone. Our data are in line with the results of bone health measurements and bone mineral density from other studies. Although we did not observe an amelioration of vitamin D levels after this micronutrient supplementation, the improvement of BUA may represent preliminary evidence of the effect of vitamin D on bone architecture that could encourage this supplementation to prevent worsening of bone structure and reduce the risk of fractures.

## Figures and Tables

**Table 1:** Demographic and clinical characteristics of patients.

Variable	OVERALL	PKU	PKU + SUP	HPA	HPA + SUP	*p*-Value
	(*n* = 110)	(*n* = 74)	(*n* = 14)	(*n* = 16)	(*n* = 6)	
Age (yr), median (IQR)	16.0 (9.0–24.0)	16.0 (9.0–24.0)	17.0 (13.5–23.5)	9.5 (7.5–19.2)	11.0 (7.0–14.3)	0.116 ^a^
Age, *n* (%)						0.204 ^b^
<18 yr	69 (62.7)	43 (58.1)	8 (57.1)	11 (68.7)	6 (100.0)	
≥18 yr	44 (37.3)	31(41.9)	6 (42.9)	5 (31.2)	-	
Gender, *n* (%)						0.326 ^b^
Female	68 (61.8)	42 (56.7)	9 (64.3)	13 (81.2)	4 (66.7)	
Male	45 (38.2)	32 (43.3)	5 (35.7)	3 (18.8)	2 (33.3)	
BMI (kg/m^2^), median (IQR)	21.5 (18.5–24.5)	21.9 (19.2–25.0)	21.9 (19.3–25.3)	21.1 (15.5–22.7)	15.8 (14.5–23.2)	0.086 ^a^

^a^ Kruskall–Wallis test; ^b^ Chi-squared test.

**Table 2:** Nutritional intakes from both amino acid substitutes and natural foods.

Variable	PKU	PKU + SUP	HPA	HPA + SUP	Mann–Whitney U Test *p*-Value
PE tot (gr)	46.7 (29.4–59.9)	43.0 (41.3–60.6)			0.762
Calcium (mg) tot	1026.4 (695.4–1196.0)	1080.5 (803.7–1227.0)			0.569
Phosphorus (mg) tot	672.5 (469.5–828.7)	747.6 (625.0–954.8)			0.366
Vitamin D (mcg) tot	18.5 (12.0–23.7)	15.9 (13.3–20.2)			0.891
gr natural protein	13.8 (10.3–18.6)	10.6 (9.38–21.8)	74.0 (49–83.00)	54.0 (53.5–56.5)	<0.001
Calcium (mg)	221 (153–337)	267 (148–365)	674 (445–699)	473 (333–684)	0.002
Phosphorus (mg)	245 (181–357)	283 (176–374)	1144 (925–1358)	927 (757–996)	<0.001
Vitamin D (mcg)	0.1 (0.07–0.19)	0.07 (0.04–0.16)	7.5 (5.77–10.4)	5.44 (3.36–7.65)	<0.001

**Table 3:** Median (interquartile range) of biochemical parameters.

Variable	PKU	PKU + SUP	HPA	HPA + SUP	Kruskall–Wallis Test *p*-Value
Calcium (9–10.8 mg/dL)	9.90 (9.50–10.10)	9.50 (9.22–9.67)	9.70 (9.30- 10.10)	9.95 (9.72–10.12)	0.176
Phenilalanine (<120 umol/L)	452.00 (314.50–764.50)	474.00 (381.00–600.25)	202.00 (115.75–242.00)	193.00 (182.50–203.50)	<0.001
Tyrosine (19–119 umol/L)	69.36 (54.13–80.83)	61.63 (58.00–79.37)	90.17 (55.50–93.54)	79.25 (68.05–87.35)	0.576
Total Protein (5.7–8.2 g/dL)	7.35 (7.00–7.70)	7.40 (7.20–7.72)	7.15 (6.97–7.25)	6.95 (6.90–7.10)	0.227
Phe/Tyr ratio (<1.3)	6.20 (4.09–14.89)	6.22 (4.21–11.96)	2.45 (1.63–3.15)	2.57 (2.48–3.48)	<0.001
25(OH)Vitamin D (30–100 ng/mL)	27.5 (22.0–38.0)	23.0 (20.5–41.3)	27.1 (20.5–31.5)	26.5 (25.0–27.3)	0.845

**Table 4:** QUS outputs among different groups.

Variable	PKU	PKU + SUP	HPA	HPA + SUP	Kruskall–Wallis Test *p*-Value
BQI	70.6 (60.4–81.2)	78.6 (71.1–87.5)	71.6 (57.6–87.3)	62.1 (47.5–79.2)	0.212
SOS	1504 (1497–1516)	1510 (1501–1517)	1502 (1495–1513)	1502 (1484–1510)	0.580
BUA	79.5 (66.2–90.8)	90.3 (85.2–92.4)	77.1 (63.6–94.6)	63.8 (60.8–81.1)	0.040
Z-SCORE	−1.47 (−2.22; −1.04)	−1.35 (−1.55; −1.10)	−0.82 (−1.20; −0.58)	n.d.	0.577
T-SCORE	−1.55 (−2.08; −1.17)	−1.13 (−2.03; −0.86)	−1.65 (−2.10; −0.71)	−1.71 (−2.41; −1.14)	0.889

n.d.: not determined.

## Data Availability

The data presented in this study are available on request from the corresponding author. (The datasets generated and/or analyzed during the current study are not publicly available due to privacy protection rules).

## Abbreviations

The following abbreviations are used in this manuscript:

HPA	Hyperphenylalaninemia
PKU	Phenylketonuria
QUS	Quantitative Ultrasound
BQI	Bone Quality Index
BUA	Broadband Ultrasound Attenuation
Phe	Phenylalanine
Tyr	Tyrosine
PAH	Phenylalanine Hydroxylase
BMD	Bone Mineral Density
AA	Amino Acids
ISCD	International Society of Clinical Densitometry
BMI	Body Mass Index
SDS	Standard Deviation Score
SOS	Speed of Sound
HPLC	High-performance Liquid Chromatography
IQR	Interquartile Range
GMP	Glycomacropeptide
CGMP	Casein Glycomacropeptide
